# Pathogen development and host responses to *Plasmopara viticola* in resistant and susceptible grapevines: an ultrastructural study

**DOI:** 10.1038/hortres.2017.33

**Published:** 2017-08-02

**Authors:** Xiao Yin, Rui-Qi Liu, Hang Su, Li Su, Yu-Rui Guo, Zi-Jia Wang, Wei Du, Mei-Jie Li, Xi Zhang, Yue-Jin Wang, Guo-Tian Liu, Yan Xu

**Affiliations:** 1 State Key Laboratory of Crop Stress Biology in Arid Areas, College of Horticulture, Northwest A&F University, Yangling, Shaanxi 712100, China; 2 College of Horticulture, Northwest A&F University, Yangling, Shaanxi 712100, China; 3 Key Laboratory of Horticultural Plant Biology and Germplasm Innovation in Northwest China, Ministry of Agriculture, College of Horticulture, Northwest A&F University, Yangling, Shaanxi 712100, China

## Abstract

The downy mildew disease in grapevines is caused by *Plasmopara viticola*. This disease poses a serious threat wherever viticulture is practiced. Wild *Vitis* species showing resistance to *P. viticola* offer a promising pathway to develop new grapevine cultivars resistant to *P. viticola* which will allow reduced use of environmentally unfriendly fungicides. Here, transmission and scanning microscopy was used to compare the resistance responses to downy mildew of three resistant genotypes of *V. davidii var. cyanocarpa*, *V. piasesezkii* and *V. pseudoreticulata* and the suceptible *V. vinifera* cultivar ‘Pinot Noir’. Following inoculation with sporangia of *P. viticola* isolate ‘YL’ on *V. vinifera* cv. ‘Pinot Noir’, the infection was characterized by a rapid spread of intercellular hyphae, a high frequency of haustorium formation within the host’s mesophyll cells, the production of sporangia and by the absence of host-cell necrosis. In contrast zoospores were collapsed in the resistant *V. pseudoreticulata* ‘Baihe-35-1’, or secretions appeared arround stomata at the beginning of the infection period in *V. davidii var. cyanocarpa* ‘Langao-5’ and *V. piasezkii* ‘Liuba-8’. The main characteristics of the resistance responses were the rapid depositions of callose and the appearance of empty hyphae and the plasmolysis of penetrated tissue. Moreover, collapsed haustoria were observed in *V. davidii var. cyanocarpa* ‘Langao-5’ at 5 days post inoculation (dpi) and in *V. piasezkii* ‘Liuba-8’ at 7 dpi. Lastly, necrosis extended beyond the zone of restricted colonization in all three resistant genotypes. Sporangia were absent in *V. piasezkii* ‘Liuba-8’ and greatly decreased in *V. davidii var. cyanocarpa* ‘Langao-5’ and in *V. pseudoreticulata* ‘Baihe-35-1’ compared with in *V. vinifera* cv. ‘Pinot Noir’. Overall, these results provide insights into the cellular biological basis of the incompatible interactions between the pathogen and the host. They indicate a number of several resistant Chinese wild species that could be used in developing new cultivars having good levels of downy mildew resistance.

## Introduction

Grapevine downy mildew is caused by the obligate biotrophic oomycete,^1^ *Plasmopara viticola* (Berk and* *Curt.) Berlese and de Toni. This disease is one of the most serious threats faced by viticulture in most areas where grapes are grown. The pathogen came from wild *Vitis* species of North America was first reported in Europe in 1878.^2^ It is believed to have been introduced to Europe on cuttings of American wild grapes, imported for use as breeding stock for *Phylloxera* resistance.^2^ Since then, it has become widespread and is now a major problem.^3^

*P. viticola* attacks all green parts of grapevines including the leaves, the clusters and young fruit.^4^ In warm, humid weather, the asexual sporangia release four to eight zoospores.^5^ When a zoospore encounters a stoma, it attaches and encysts. Next it forms a germ tube that penetrates the substomatal cavity. Subsequently, this germ tube swells into an infection vesicle.^6^ A primary hypha appears from an infection vesicle and quickly develops branches and haustoria.^7^ After an incubation period of several days (sometimes in as few as 4 days),^5^ sporangiophores emerge through the stomatum and form sporangia.^8^ At the end of autumn, numerous oospores form within fallen leaves and berries allowing *P. viticola* to overwinter.^9^

It has been reported that *Vitis* species and cultivars vary in resistance to *P. viticola*.^10,11,12^ Generally, *V. vinifera* cultivars such as ‘Pinot noir’ are susceptible to *P. viticola* while most *Vitis* species from North America are highly resistant,^13^ for example, *V. rupestris* and *V. riparia*,^14^ while *Muscadinia rotundifolia* is immune.^15^ Some Chinese wild *Vitis* genotypes such as *V. pseudoreticulata* ‘Baihe-35-1’ and *V. davidii var. cyanocarpa* ‘Langao-5’ are also resistant to *P. viticola*, while *V. piasezkii* ‘Liuba-8’ is highly resistant.^16,17^

Many studies have reported on the mechanisms of resistance against *P. viticola* infection in grapevine at the histological and ultrastructural levels, including callose deposition in stomata,^16,18^ lignification,^19^ stilbenic phytoalexin production,^20,21^ hydrogen peroxide (H_2_O_2_) accumulation ^22,23^ and hypersensitive reactions.^24^ The ultrastructural response to downy mildew varies among the resistant species. Using KOH-aniline blue fluorescent staining, Gindro *et al.* observed callose and secretions in the resistant *V. vinifera* cv. ‘Solaris’ but not in the susceptible *V. vinifera* cv. ‘Chasselas’.^8^ Haustoria and hyphae were found to be degenerated in *V. riparia* (var. Gloire de Montpellier) and *M. rotundifolia* cv. ‘Carlos’^10,25^ using transmission electron microscopy and these reactions may reduce pathogen growth.^26^ In addition, trichomes and bristles, cuticular waxes, density of stomata and internal cuticular rims may be related to resistance to *P. viticola*.^27,28^ In a previous study, the reaction of Chinese wild *Vitis* species to *P. viticola* was elucidated at the histological level.^16^ In this study we aim to compare, at the ultrastructural level, the characterization of the pathogen development and host response during incompatible and compatible interactions between isolate *P. viticola* ‘YL’ and three resistant Chinese wild *Vitis* species and one susceptible *V. vinifera* cultivar.

## Materials and methods

### Pathogen and inoculation

The *P. viticola* isolate ‘YL’ (Yang Ling town) was collected from an infected grapevine leave (‘011’, hybrid of *V. vinifera*×*V. riparia*) in the Grape Repository of Northwest A&F University, Yangling, Shaanxi, China. The method for isolating *P. viticola* ‘YL’ refers to Wong and Wilcox.^29^ Briefly, *P. viticola* was purified for three times by single sporangiophore transfer from infected leaves, then the isolate was propagated weekly on detached leaves of *V. vinifera* cv. ‘Pinot Noir’ in 90 mm Petri dishes (abaxial surface upwards) on wet filter paper and incubated in a controlled environment under a photoperiod (temperature) of 16 h light (22 °C) and 8 h of darkness (18 °C) and 80% relative humidity.

The third and fourth fully-expanded leaves from the apex of *V. vinifera* cv. ‘Pinot Noir’, *V. pseudoreticulata* ‘Baihe-35-1’, *V. davidii var. cyanocarpa* ‘Langao-5’ and *V. piasezkii* ‘Liuba-8’ were obtained from the Grape Repository of Northwest A&F University, Yangling, Shaanxi, China.^16^ The leaf surfaces were sterilized using bleach (0.01%) and then rinsed three times in sterile distilled water. Leaf discs (10 mm diameter) were obtained with a cork borer. The abaxial surfaces were inoculated with 50 μl drops of an aqueous suspension of 5×10^4^ sporangia per ml and placed on wet filter paper in 90 mm Petri dishes. The control groups of leaf discs were inoculated with 50 μl drops of sterile distilled water. Incubation conditions were as described above.

### Scanning electron microscopy

Samples of leaf discs were collected at 0.5 day post inoculation (dpi), and at 1, 3, 4, 5, 6, 7, 8, 9, 10 and 11 dpi. Samples were cut into 5×5 mm pieces, fixed in 4% glutaraldehyde at 4 °C overnight, rinsed four times with 0.1 m phosphate buffered saline (pH 6.8) for 10 min each and dehydrated in a graded series of aqueous ethanol solutions (10, 30, 50, 70, 80, 90, 100, 100% ethanol)—each step for 15 min. Finally, samples were soaked in isoamyl acetate for 30 min, critical point dried in CO_2_ and coated with gold. Mounted samples were viewed with an Hitachi S-4800 (Ibaraki, Japan) scanning electron microscope at 10 kV.

### Transmission electron microscopy

Samples (2×5 mm) of leaf discs were taken at 3, 5 and 7 dpi, fixed immediately in 4% glutaraldehyde at 4 °C overnight and washed with 0.1 m phosphate buffered saline (pH 6.8) four times for 10 min each. The samples were then fixed in 1% osmium tetroxide (OsO_4_) for 2 h, dehydrated in a graded series of ethanol (as above), and infiltrated with London Resin Company Ltd (LR) White resin (Basingstoke, UK). This involved infiltration in a series of mixtures of acetone and LR White resin in the proportions, 3:1, 1:1 and 1:3 (vol/vol) for 6 h each, then a final infiltration in pure resin for 72 h. Finally, the samples were embedded in pure LR White resin and polymerized at 55 °C for 48 h.

Semi-thin sections (1 μm) of samples were cut with a glass knife and stained with 0.3% aqueous toluidine blue in 1.89% sodium tetraborate. Semi-thin sections were examined in bright-field microscopy using an Olympus BX 51 (Tokyo, Japan) to focus on infected sites. Ultra-thin sections (90 nm) were cut with a diamond knife, collected on copper grids, stained with uranyl acetate and lead citrate and observed by transmission electron microscopy 1230 (JEOL) (JEM-1230, Tokyo, Japan) at 80 kV.

### Test of sporangial density and percentage of infected stomata

Twelve leaf discs were inoculated as described above. At 9 dpi, sporangia on each leaf disc were shaken into a 2 ml plastic tube containing 1 ml of distilled water. The number of sporangia in the water was measured by turbidimetry with a spectrophotometer at 400 nm by the method of Gindro and Pezet.^30^ Aqueous suspensions of 5×10^4^ sporangia per ml were confirmed using a hemocytometer for calibration.

At 1 dpi, 12 leaf discs were stained with KOH-aniline blue by the procedure of Díez-Navajas *et al.*^31^ The percentage of infected stomata (number of stomata with substomatal vesicles per total number of stomata observed) was calculated according to Yu *et al.*^7^ using a fluorescence microscope (Olympus BX-51, with excitation wavelength 400–440 nm and emission wavelength 475 nm).^16^

### Data analysis

Each experiment was carried out in triplicate. Data were analyzed by analysis of variance to detect any statistically significant differences. Least significant differences are reported at *P*⩽0.05. All calculations were carried out using SPSS 13.0 software (Chicago, IL, USA).

## Results

### Cytological differences in *Plasmopara viticola* isolate ‘YL’ infected grape leaves

**Figure 1:**
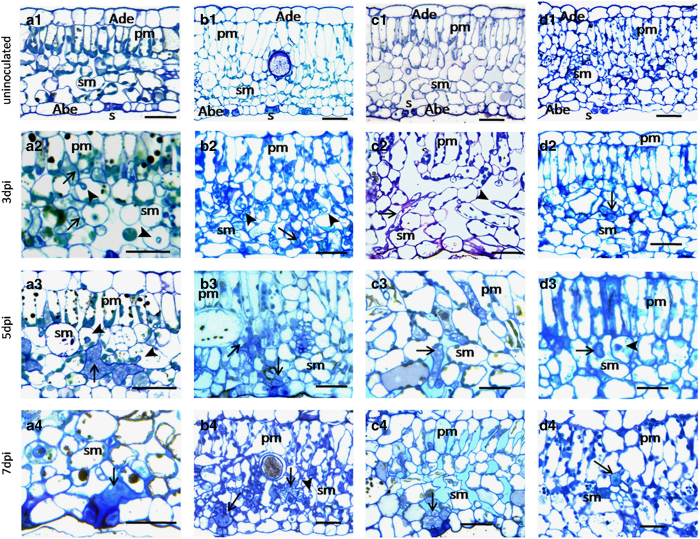
Cytological study of *Plasmopara viticola* isolate ‘YL’ infected grape leaves. *Vitis vinifera* cv. ‘Pinot Noir’ (**a**); *V. pseudoreticulata* ‘Baihe-35-1’ (**b**); *V. davidii var. cyanocarpa* ‘Langao-5’ (**c**); *V. piasezkii* ‘Liuba-8’ (**d**). Un-inoculated with *P. viticola* (**a**1–**d**1); 3 days post inoculation (dpi) (**a**2–**d**2); at 5 dpi (**a**3–**d**3) and at 7 dpi (**a**3–**d**3). Abe, abaxial epidermis; Ade, adaxial epidermis; arrow, hyphae; arrow head, haustorium; pm, palisade mesophyll; s, stoma; sm, spongy mesophyll. Scale bar, 25 μm.

Initially, we used bright-field microscopy for cytological observation. The hyphae stained with toluidine blue showed vacuolation and contrasting colors of the cell wall and cytoplasm in all samples. In ‘Pinot Noir’ at 3 dpi, hyphae were widespread and closely attached to the cell wall of mesophyll cells and filled the intercellular spaces of the leaves. A high frequency of pyriform haustoria was observed, breaking through the mesophyll cell walls and with some haustoria reaching the palisade mesophyll (Figure 1a2). In ‘Baihe-35-1’ and ‘Langao-5’, hyphae were small and haustoria were less common than in ‘Pinot Noir’ at 3 dpi (Figures 1b2 and c2). At 5 dpi, in ‘Pinot Noir’, hyphae were extensive and most mesophyll tissues were colonized (Figure 1a3). However, no obvious change was observed in ‘Baihe-35-1’ and ‘Langao-5’ (Figures 1b3 and c3). At 7 dpi, in ‘Pinot Noir’, ‘Baihe-35-1’ and ‘Langao-5’ hyphae accumulated in the substomatal cavities (Figure 1a4). In ‘Liuba-8’, hyphae were small and strongly restricted without any change from 3 to 7 dpi (Figures 1d2–d4). These observations suggest the hyphal growth in the three resistant genotypes were inhibited.

### Ultrastructural study of *Plasmopara viticola* isolate ‘YL’ infection in the four genotypes

**Figure 2:**
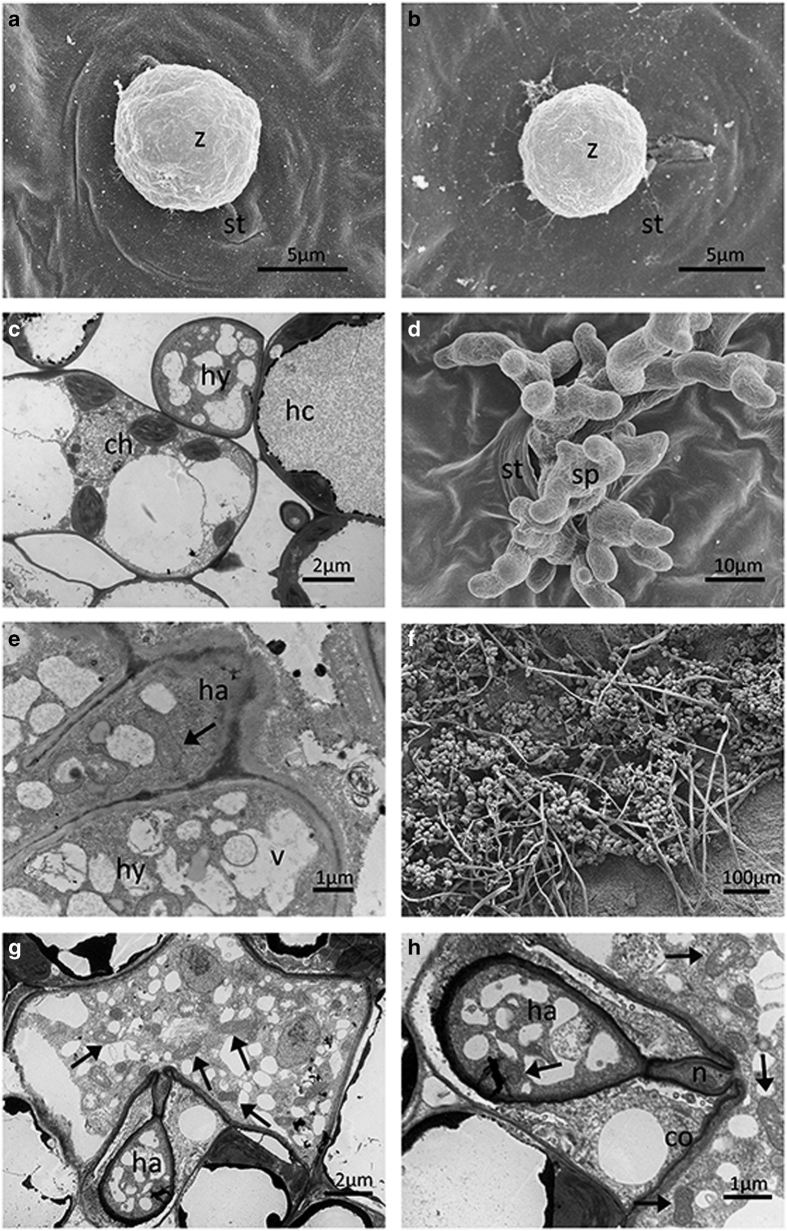
Ultrastructural study of *Plasmopara viticola* isolate ‘YL’ infection on leaves of *Vitis vinifera* cv. ‘Pinot Noir’. Encysted zoospore at half-a-day post inoculation (dpi) (**a**). Encysted zoospore at 1 dpi (**b**). Normal hyphae at 3 dpi (**c**). New sporangiophores emerging through stoma at 4 dpi (**d**). Normal hyphae and haustoria at 5 dpi (**e**). Fully developed sporangiophores and sporangia at 5 dpi (**f**). Pyriform haustoria and hyphae with numerous mitochondria (arrow) at 7 dpi (**g**). (**h**) Detail of (**g**). ch, chloroplast; co, collar; hc, host cell; hy, hyphae; ha, haustorium; n, neck; s, stomata; sp, sporangiophores; v, vesicle; z, encysted zoospore.

**Figure 3:**
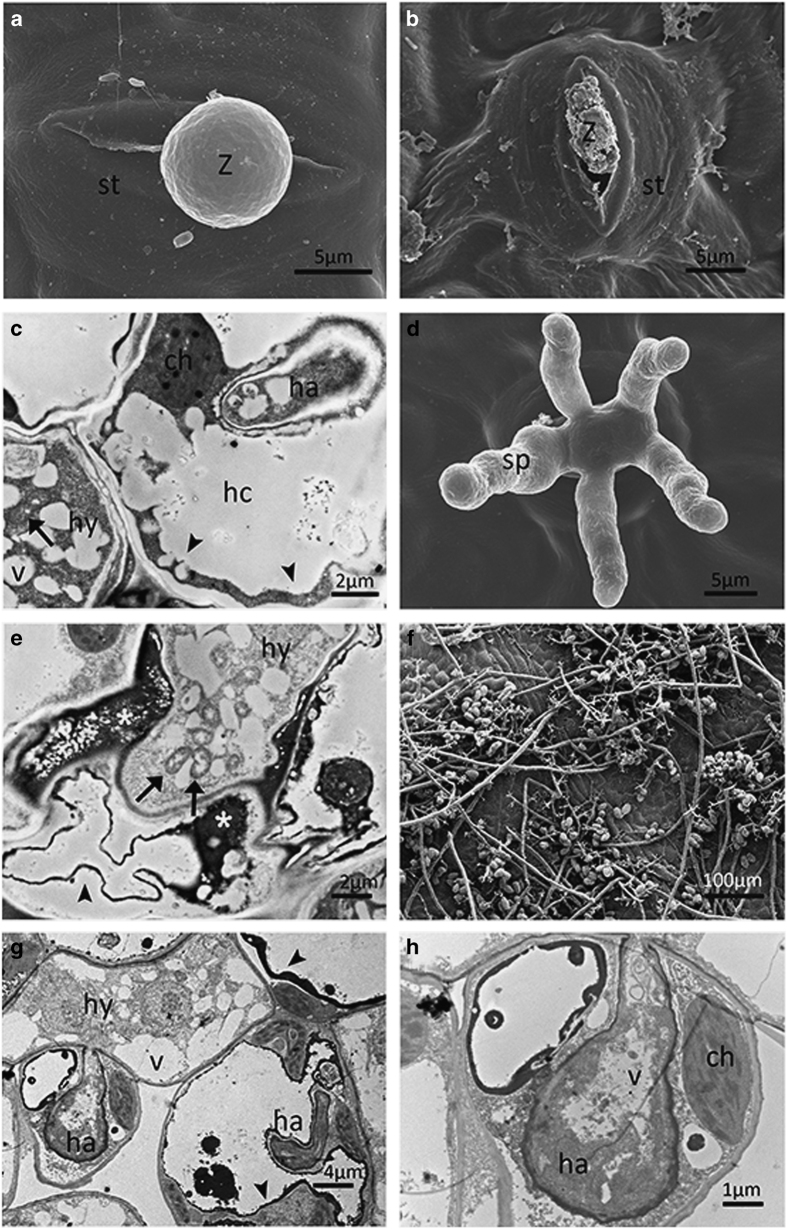
Ultrastructural study of *Plasmopara viticola* isolate ‘YL’ infection on *Vitis pseudoreticulata* ‘Baihe-35-1’. Encysted zoospore at half-a-day post inoculation (dpi) (**a**). Putatively collapsed zoospores at 1 dpi (**b**). Plasmolysis (arrow head) in host cell at 3 dpi (**c**). New sporangiophores emerging through stomata at 5 dpi (**d**). Higher electron-dense material (white asterisks) and plasmolysis (arrow head) in host cell at 5 dpi (**e**). Fully developed sporangiophores and sporangia at 6 dpi (**f**). Extensively vacuolated hyphae and haustoria at 7 dpi (**g**). (**h**) Detail of (**g**). arrow, mitochondria; ch, chloroplast;ha, haustorium; hc, host cell; hy, hyphae; s, stomata; v, vesicle; z, encysted zoospore.

**Figure 4:**
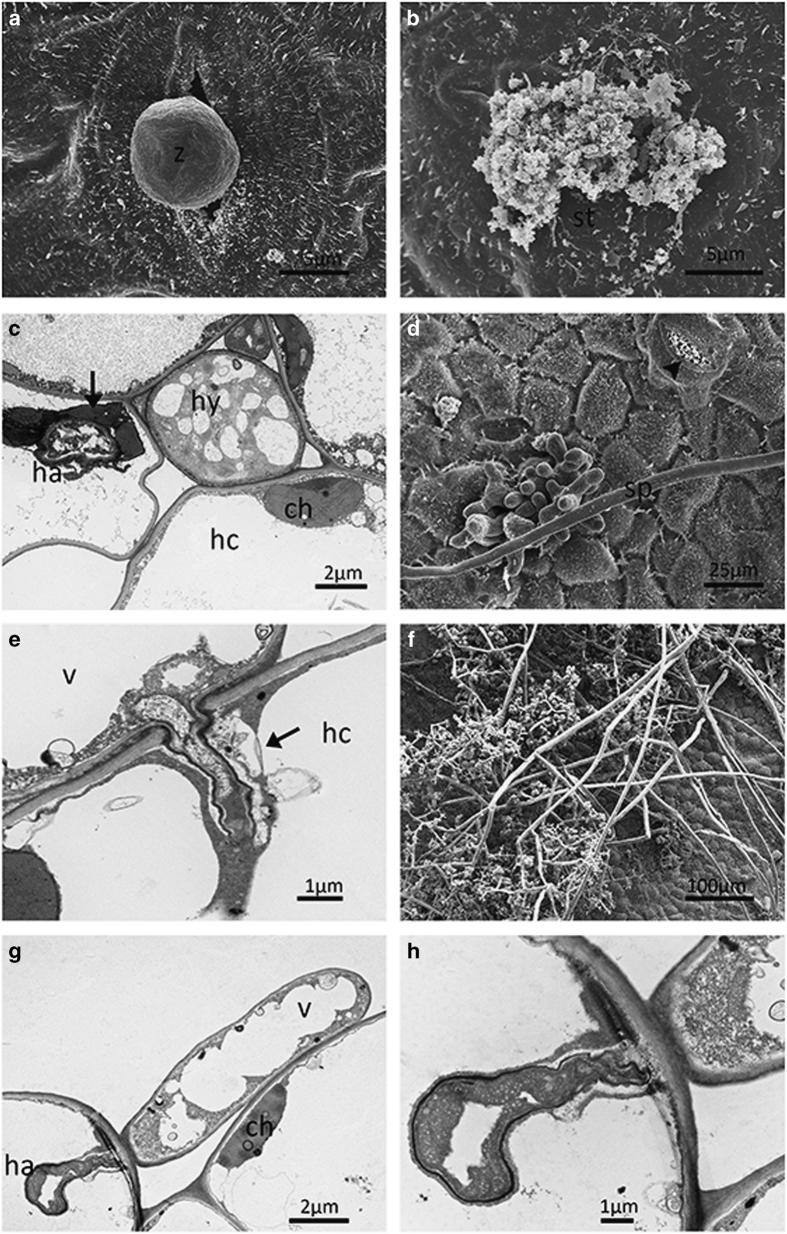
Ultrastructural study of *Plasmopara viticola* isolate ‘YL’ infection on *Vitis davidii var. cyanocarpa* ‘Langao-5’. Encysted zoospore at half-a-day post inoculation (dpi) (**a**). Secretions in stomata at 1 dpi (**b**). Irregular haustoria encased with amorphous material (arrow) at 3 dpi (**c**). New sporangiophores emerging through stomata and secretions (arrow head) in stomata at 5 dpi (**d**). Collapsed haustoria and extensively vacuolated hyphae (**e**). Fully developed sporangiophores and sporangia at 6 dpi (**f**). Extensively vacuolated hyphae and irregular haustoria with a distinct narrow, dark wall at 7 dpi (**h**). (**h**) Detail of (**g**). ch, chloroplast; ha, haustorium; hc, host cell; hy, hyphae; s, stomata; v, vesicle; z, encysted zoospore.

**Figure 5:**
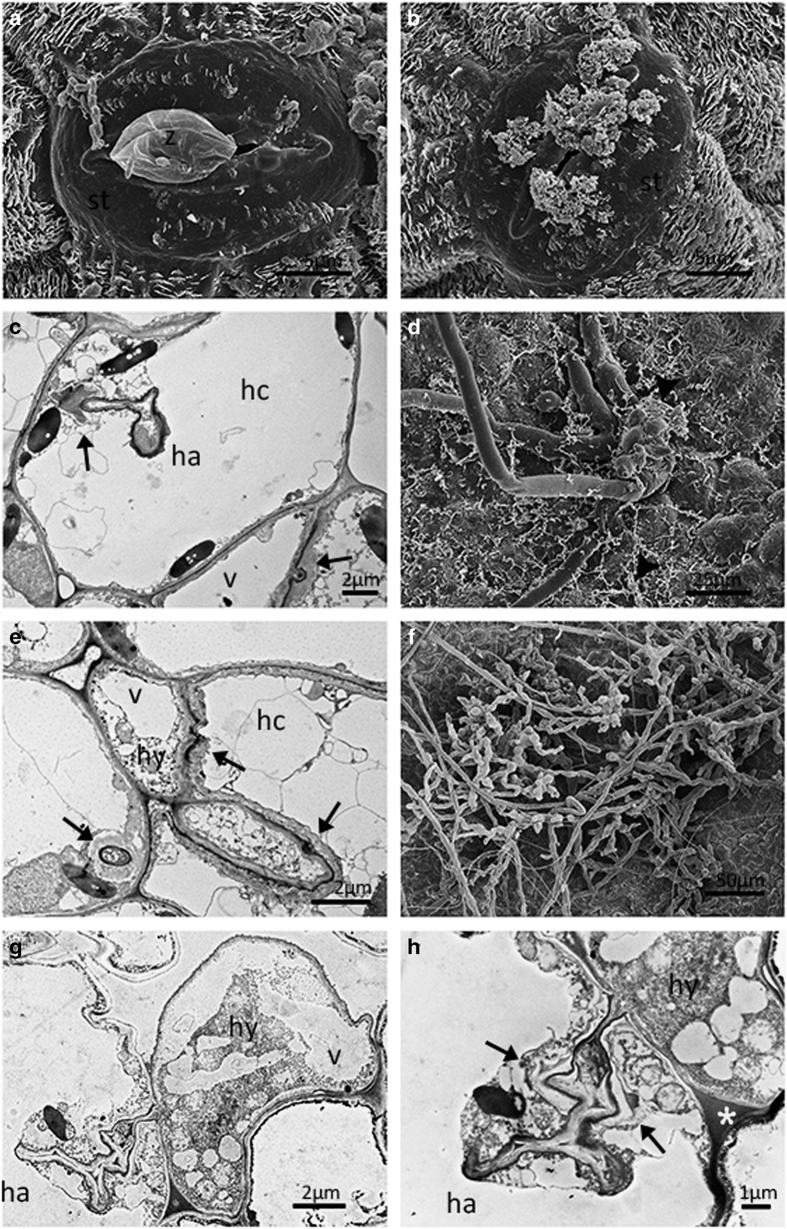
Ultrastructural study of *Plasmopara viticola* isolate ‘YL’ infection on *Vitis piasezkii* ‘Liuba-8’. Encysted zoospore at half-a-day post inoculation (dpi) (**a**). Secretions in stomata at 1 dpi (**b**). Irregular haustoria encased with amorphous material (arrow) at 3 dpi (**c**). New sporangiophores emerging through stomata and secretions (arrow head) around sporangiophores at 10 dpi (**d**). Extensively vacuolated hyphae with amorphous electron-dense material between host cell and hyphae at 5 dpi (**e**). Sterile sporangiophores without sporangia developed at 11 dpi (**f**). Extensively vacuolated hyphae and damaged haustoria presumably surrounded by callose (arrow) at 7 dpi (**g**). (**h**) Detail of (**g**). arrow head, secretions; asterisks, amorphous electron-dense material; ch, chloroplast; ha, haustorium; hc, host cell; hy, hyphae; s, stomata; v, vesicle; z, encysted zoospore.

To provide a more comprehensive insight, we carried out an ultrastructural study. At 0.5 dpi, encystment of zoospores was observed on the stomata of all genotypes (Figures 2a–5aFigure 5). At 1 dpi, no host reactions were observed in ‘Pinot Noir’ (Figure 2b), whereas structures of zoospores were collapsed in ‘Baihe-35-1’ (Figure 3b) and stomata surrounded with secretions appeared in ‘Langao-5’ and ‘Liuba-8’ (Figures 4b and 5b).

At 3 dpi, hyphae in ‘Pinot Noir’ were characterized by normal ultrastructure with numerous vacuoles (Figure 2c). In ‘Baihe-35-1’, mitochondria were observed in the hyphae that were also full of vacuoles, while plasmolysis occurred in the host cells (Figure 3c). In ‘Liuba-8’ and ‘Langao-5’, haustoria were irregular and encased in an amorphous material, presumably callose (Figures 4c and 5c).

At 5 dpi, in ‘Pinot Noir’, the cytoplasmic membrane of hyphae and haustoria were closely attached to the cell wall of mesophyll cells and with homogenous cell of the leaves (Figure 2e). In ‘Baihe-35-1’, the cell wall of any mesophyll cell in contact with a hypha showed high electron density. Mitochondria were observed and hyphae were full of vacuoles and host tissue were plasmolyzed (Figure 3e). In ‘Langao-5’, highly vacuolated hyphae and collapsed haustoria were observed (Figure 4e). In ‘Liuba-8’, hyphae were extensively vacuolated and haustoria were surrounded with an amorphous, electron-dense material, presumably callose (Figure 5e).

At 7 dpi, in ‘Pinot Noir’ typically pyriform haustoria showing the accumulation of electron-dense material, continuous with the extra-haustorial matrix were observed in host cells and the hyphae were characterized by the presence of numerous mitochondria and vacuoles (Figures 2g and h). In ‘Baihe-35-1’, host cells were plasmolyzed (Figure 3h) and highly vacuolated hyphae and haustoria were also observed (Figure 3g). In ‘Langao-5’ hyphae were partially vacuolated (Figure 4h). Irregular haustoria with distinct narrow dark walls were observed (Figure 4g). Haustoria in ‘Liuba-8’ were severely degenerate and encased, presumably, with callose (Figure 5h). In addition, hyphae were highly vacuolated and without recognizable mitochondria (Figure 5g).

In ‘Pinot Noir’ and ‘Baihe-35-1’, new sporangiophores emerged through the stomata at 4 and 5 dpi, respectively, (Figures 2d and 3d). In contrast secretions in stomata near the new sporangiophores were observed at 5 dpi in ‘Langao-5’ (Figure 4d). However, in ‘Liuba-8’, new sporangiophores did not emerge until 10 dpi. Secretions were also observed around the sporangiophores (Figure 5d). This suggests the infection progress of hyphae was much delayed in ‘Liuba-8’, compared with the other three grapes.

Sporangiophores and sporangia were fully developed in ‘Pinot Noir’ at 5 dpi (Figure 2f). For ‘Langao-5’ and ‘Baihe-35-1’ sporangia appeared at 6 dpi (Figures 3f and 4f). However, in ‘Liuba-8’ sterile sporangiophores without sporangia appeared at 11 dpi (Figure 5f).

### Sporangial density and percentage of infected stomata

**Figure 6:**
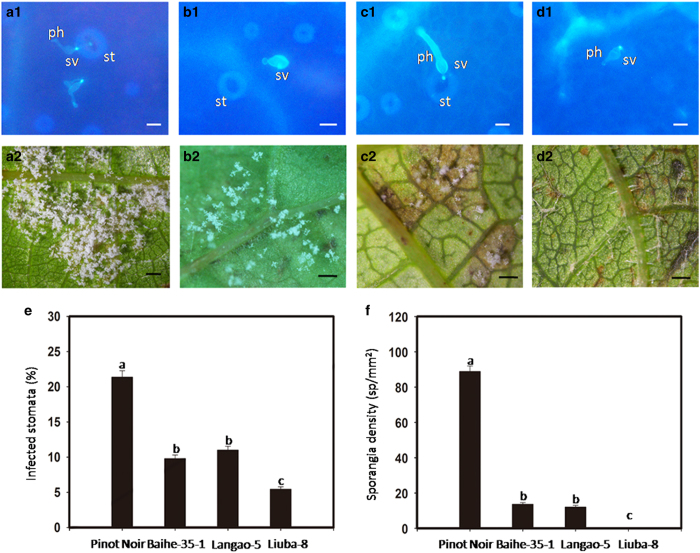
Sporangial density and percentage of *Plasmopara viticola* isolate ‘YL’ infected stomata. *Vitis vinifera* cv.‘Pinot Noir’ (**a**); *V. pseudoreticulata* ‘Baihe-35-1’ (**b**); *V. davidii var. cyanocarpa* ‘Langao-5’ (**c**); *V. piasezkii* ‘Liuba-8’ (**d**). Infected stomata with substomatal vesicles stained by aniline blue and observed under fluorescence microscope at 1 day post inoculation (dpi) (**a**1-**d**1). sv, substomatal vesicles; st, stomata; ph, primary hyphae. Scale bar, 10 μm. Observations on leaf discs of four genotypes at 9 dpi. Scale bar, 200 μm. Percentage of infected stomata in four genotypes at 1 dpi (**e**). Sporangial density was tested on four genotypes at 9 dpi (**f**). Error bars represent standard errors. Data were analyzed according to a least significant difference test *P*⩽0.05. Different letters indicate significantly different values (*n*=30).

At 1 dpi, infected stomata with substomatal vesicles were observed in all genotypes (Figures 6a1–d1). The percentage of infected stomata in ‘Pinot Noir’ (21.66%) was higher than that in ‘Baihe-35-1’ (9.81%), ‘Langao-5’ (11.09%) and much higher than in ‘Liuba-8’ (5.53%) (Figure 6e).

At 9 dpi, a high sporulation density was observed on ‘Pinot Noir’ under the stereomicroscope (Figure 6a2). Low sporulation densities and necrotic spots appeared on ‘Baihe-35-1’ and ‘Langao-5’ (Figures 6b2 and c2). No sporulation was seen in ‘Liuba-8’ but necrotic spots could be seen (Figure 6d2). Sporangial density was assessed by spectrophotometry. The average sporangial density on ‘Pinot Noir’ was 88.86 sporangia/mm^2^, on ‘Baihe-35-1’ it was 13.67 sporangia per mm^2^ and on ‘Langao-5’ it was 12.10 sporangia per mm^2^ (Figure 6f). No sporangia could be detected on ‘Liuba-8’ by spectrophotometry (Figure 6f).

## Discussion

Previous studies have shown that field populations of *P. viticola* may comprise several genotypes.^32^ Although field isolates of *P. viticola* have typically been used to evaluate resistance levels in grapevines, this procedure prevents a rigorous comparative evaluation of the interactions between host and pathogen genotypes.^19^ Instead, we used here, a purified isolate of *P. viticola* ‘YL’ to eliminate putative effects due to pathogen heterogeneity.

Stomata has key roles during *P. viticola* infections. Zoospores of *P. viticola* encyst near stomata and new sporangiophores emerge through stomata.^8^ In the resistant *V. vinifera* cv. ‘Solaris’, a rapid defense response is observed (scanning electron microscope) when germ tubes of encysted zoospores penetrate stomata.^7,8^ However, for the partially resistant genotypes, a defense response appears only when the first haustoria have contacted the mesophyll cells.^33^ In this study, zoospores were collapsed in ‘Baihe-35-1’ and stomata surrounded with secretions appeared in ‘Langao-5’ and ‘Liuba-8’ at 1 dpi. This would seem to indicate that the restriction of *P. viticola* occurred very rapidly—within 24 h. This observation is similar to that reported by Gindro *et al.* and Yu *et al.*^7,8^ The key stages to which to distinguish resistance and susceptibilty to *P. viticola* are zoospore infection and haustorial formation.^7^ At 1 dpi, substomatal vesicles with primary hyphae were observed in both susceptible and resistant genotypes.^5,33^ After 1 dpi, the hyphae of *P. viticola* expand rapidly and branch in the susceptible genotypes, while in the resistant genotypes, most hyphae branch slowly or remain unbranched.^5^ In this study, *P. viticola* isolate ‘YL’ successfully infected both susceptible and resistant genotypes which further supports earlier findings.^5,7,16,33^ However, the percentage of infected stomata and the scanning electron microscope observations both indicate that the infection rate of zoospores was significantly reduced in ‘Baihe-35-1’ and ‘Langao-5’ and greatly reduced in ‘Liuba-8’.

Callose deposition is a host-cell defense mechanism^34^ and has been reported in resistance to many fungi, such as to rust, downy mildew and powdery mildew.^35,36,37^ It is thought that callose deposition may limit or impede pathogen growth and block nutrient uptake from the host cell by encasing haustoria.^19,34^ In our study, callose material was not observed in ‘Pinot Noir’; however, callose deposition was observed in the three resistant genotypes. The growth of *P. viticola* hyphae was slowed down so that the times required by the sporangiophores to emerge were increased and the sporangial densities in the three wild genotypes were reduced. These results are consistent with those of previous studies.^8,16,38^

Empty hyphae or enlarged vacuoles have been observed in older hyphae including haustoria while young hyphae have relatively few vacuoles.^25^ Extensive vacuolation and degenerate hyphae with deposits of electron-dense material are the result of altered biosynthesis in the pathogen cell wall after treatment with the fungicide Mandipropamid.^26^ Similar results have also been described in *P. viticola* after treatment with diketopiperazines extracted from *Alternaria alernata* ^39^ and in *P. viticola* coupled with viral infection.^40^ In ‘Langao-5’ and ‘Liuba-8’, empty hyphae were observed as early as 5 dpi while in ‘Baihe-35-1’ enlarged vacuoles were observed in hyphae and haustoria at 7 dpi, indicating the hyphae were degenerate. However, in ‘Pinot Noir’, the numerous mitochondria observed in hyphae and haustoria were covered with an extra-haustorial matrix by 7 dpi, indicating that the pathogen was viable and had high metabolic activity.^41^

Mature haustoria may function as a site of molecular exchange of effectors and nutrients between the host cell and pathogen.^42^ Abnormal or collapsed haustoria have been reported in incompatible interactions between *Arabidopsis thaliana* and *Peronospora parasitica*,^34^ resistant cv. ‘IRAC 2091’ (Gamaret×Bronner) interaction with *P. viticola*^10^ and *P. viticola* infected grapevine leaves after treatment with diketopiperazine extracts^39^ or sulfated laminarin (PS3).^43,44^ Collapsed haustoria were observed in ‘Langao-5’ at 5 dpi and in ‘Liuba-8’ at 7 dpi which is consistent with the earlier research, referred to above. This suggests these haustoria are unable to take up nutrients from the host cell.

Previous studies have shown that plasmolysis occurs in *P. viticola* structures when grapevine leaves are treated with aqueous solutions of Cu(OH)_2 _(ref. 39) or Mandipropamid.^26^ Plasma membranes of hyphae detached from the cell wall were observed in ‘Langao-5’ at 7 dpi which also showed extended lysis according to Toffolatti.^26^ Plasmolysis was observed in ‘Langao-5’ at 7 dpi and in ‘Baihe-35-1’ at 5 dpi and 7 dpi which is presumably a response by the host cell when exposed to hyperosmotic stress.^45^

None of the Chinese wild *Vitis* species, including the three genotypes mentioned above, showed complete immunity to *P. viticola*.^46^ In addition, ‘Liuba-8’ and *V. labrusca* ‘Beaumont’ exhibited the same level (highly resistant) of resistance to *P. viticola* according to Wan *et al.*^17^ In this study, although the sterile sporangiophores emerged at 11 dpi, sporangia were not observed in the highly resistant genotype ‘Liuba-8’. This shows that the pathogen cannot complete its life cycle in ‘Liuba-8’. A similar effect was described in resistant *V. rupestris* 66 h post inoculation, when unbranched and long sterile hyphae emerged from stomata.^5^ Also, necrotic spots were evident to the naked eye, on the leaves of three wild genotypes which is consistent with Liu *et al.*,^16^ and could be associated with a hypersensitive reaction.^24^ The necrotic zone may prevent hyphal extension. Evaluation of sporangial density has been widely used for estimation of resistance to *P. viticola* in grapevines.^10,38,47^ Production of sporangia can be the source of secondary infections. In this study, sporangia were not observed or detected in ‘Liuba-8’ inferring that the chances of secondary infection in ‘Liuba-8’ were very much reduced. In ‘Baihe-35-1’ and ‘Langao-5’, the resistance reactions only delayed the growth of the pathogen and sporangia can be detected but numbers were limited, compared with in ‘Pinot Noir’.

Ampelographic characteristics such as erect and reclining trichomes, inner cuticular rims, waxes, structure of epidermis and mesophyll and stomatal density can act as physical barriers to zoospore infection at early stages.^27,28,48,49^ In this study, no clear relationships between susceptibility to *P. viticola* isolate ‘YL’ and these ampelographic characters could be found (Supplementary Figure S1 and Supplementary Table S1), which is consistent with early studies.^19,27,48^

Differences in response between resistant and susceptible grapevine genotypes to *P. viticola* isolate ‘YL’ were: irregular haustoria, empty hyphae, callose depositions and plasmolysis in resistant host cells. These results provide insights into the mechanisms of resistance in a number of resistant Chinese wild genotypes. These have important potential as germplasm resources having resistance to *P. viticola* and have possible value in the development of new, *P. viticola* resistant, grape cultivars. Although the mechanisms of inhibition of *P. viticola* development in resistant grapevines remain not fully understood, further work will focus on the molecular mechanisms of these responses and on identifying resistance genes in these genotypes.
