# Supplementary material for: Pathogen development and host responses to Plasmopara viticola in resistant and susceptible grapevines: an ultrastructural study
Source: Hortic Res. 2017 Aug 2;4:17033–. doi: 10.1038/hortres.2017.33 (PMC5539432; doi:10.1038/hortres.2017.33)
Supplement: Supplementary Information [file hortres201733-s1.doc]

**Supplementary material**

**Ampelographic characteristics**

Narrow palisade mesophyll with 4-5 layers of spongy mesophyll were observed in four genotypes. *V. pseudoreticulata ‘*Baihe-35-1’ and *V. piasezkii* ‘Liuba-8’ had more tightly packed cells with small intercellulare space (Fig. S1 B1 and D1), whereas in *V. davidii var. cyanocarpa* ‘Langao-5’ and *V. vinifera* cv ‘Pinot noir’ it were loosen (Fig. S1 A1 and C1). Table 1 showed the values of ampelographic differences between four genotypes. *V. davidii var. cyanocarpa* ‘Langao-5’ has the thickest spongy mesophyll (80.08 ± 6.92μm), abaxial epidermis (13.82 ± 1.41μm) and whole leaf thickness (142.90 ± 11.13μm) but the lowest (20.13 ± 3.40/visible field). *V. pseudoreticulata ‘*Baihe-35-1’ has the thickest adaxial epidermis (16.12 ± 1.40μm) and the highest stomatal density (31.90 ± 3.29/visible field) while *V. piasezkii* ‘Liuba-8’ has the thickest palisade mesophyll (44.49 ± 3.46μm). *V. vinifera* cv Pinot noir has the lowest adaxial epidermis (12.96 ± 1.64μm), abaxial epidermis (8.58 ± 1.37μm), palisade mesophyll (21.34 ± 3.14μm), spongy mesophyll (39.14 ± 3.62μm) and whole leaf thickness (82.54 ± 6.18μm).

In *V. vinifera* cv Pinot noir, cuticular showed in the adaxal epidermal cell and reclining hairs on the abaxial epidermal (Fig. S1 A3-4). Wax deposits were observed on both sides of epidermis in *V. davidii var. cyanocarpa* ‘Langao-5’ (Fig. S1 C3-C4) and *V. piasezkii* ‘Liuba-8’ (Fig. 8 D3-D4). Reclining and erect hairs were on the both both sides of epidermis in *V. piasezkii* ‘Liuba-8’ (Fig. 8 D3-D4). *V. pseudoreticulata ‘*Baihe-35-1’ (Fig. S1 B3-B4) and *V. davidii var. cyanocarpa* ‘Langao-5’ (Fig. S1 C3-C4) don`t have hairs.

**Fig S1. Ampelographic characteristics of differences epidermises.**

*V. vinifera* cv ‘Pinot noir’ (A); *V. pseudoreticulata ‘*Baihe-35-1’ (B); *V. davidii var. cyanocarpa* ‘Langao-5’ (C); *V. piasezkii* ‘Liuba-8’ (D). Macroscopic observations on adaxial epidermis (A1-D1)；Macroscopic observations on abaxial epidermis(A2-D2)； Scanning electron microscopy of adaxial epidermis (A3-D3); Scanning electron microscopy of abaxial epidermis (A4-D4); Stomata (A5-D5)

**Table S1 Comparison of average leaves in terms of leaf variables**.

^w^ Thicknesses. μm.

^x^ Stomatal density：number per 0.139 mm^2^ (visible field)

^y^ Hairs of abaxial epidermis

^z^ According to Liu et al.[^1^](#_ENREF_1)

The data correspond to values ± standard deviation and values with the same letter are not significantly different (P ≤0.05). (n=30)

**Fig S1.**

**
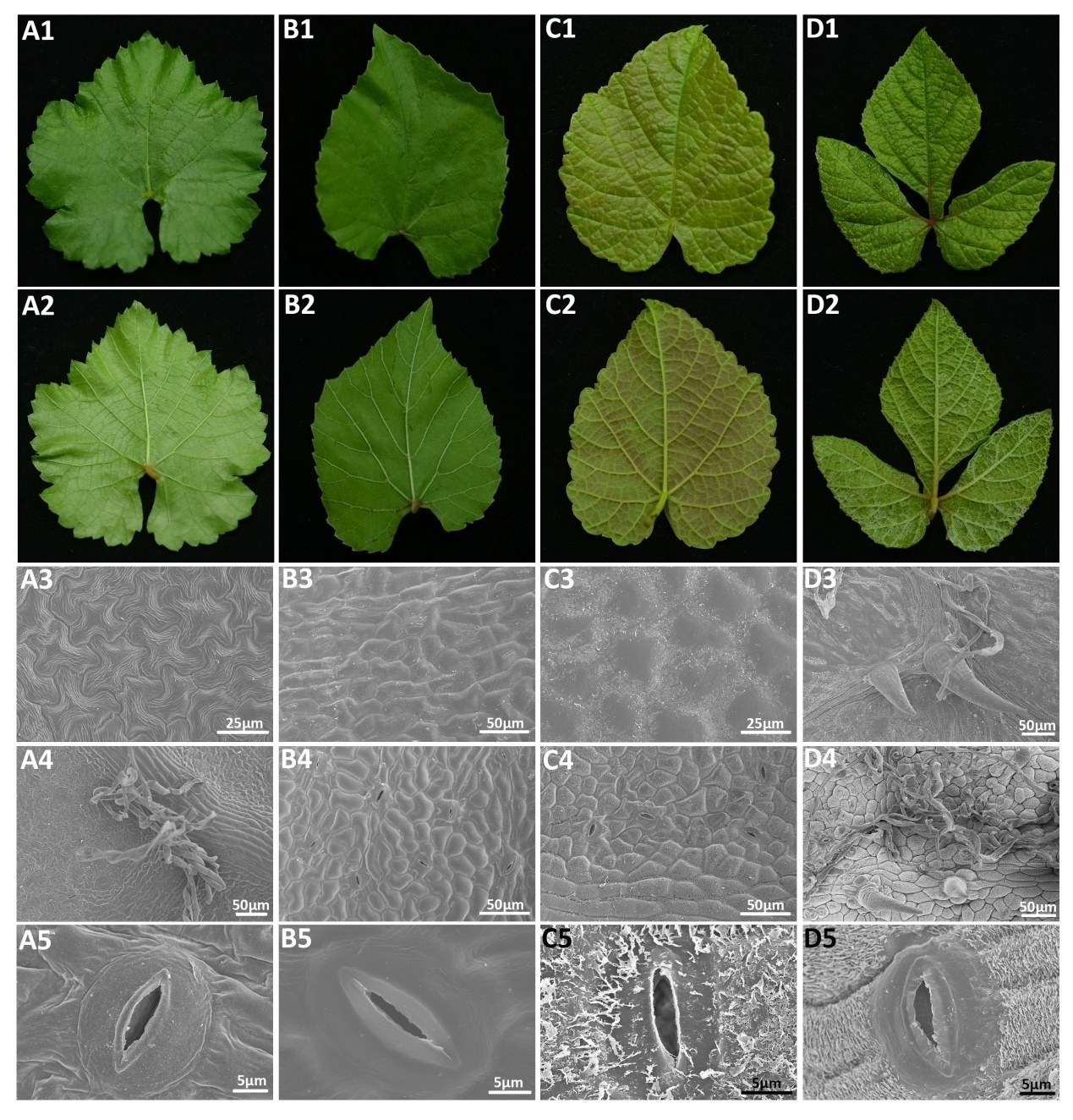
**

**Table S**1

| **Genotypes** | **Adaxial** | **Abaxial** | **Palisade** | **Spongy** | **Whole leaf** | **Stomatal** | **Waxes**^y^ | **Hairs** ^y^ | **Resistant** |
| --- | --- | --- | --- | --- | --- | --- | --- | --- | --- |
|  | **epidermis** ^w^ | **epidermis** ^w^ | **mesophyll** ^w^ | **mesophyll**^w^ | **thicknesses** ^w^ | **density** ^x^ |  |  | **levels** ^z^ |
| *V. vinifera cv. Pinot noir* | 12.96 ±1.64c | 8.58±1.37c | 21.34±3.14d | 39.14±3.62d | 82.54±6.18c | 23.76±3.75c | - | + | S |
| *V. Pseudoreticulaia* Baihe-35-1 | 16.12±1.40a | 11.35±2.12b | 38.30±2.46c | 49.69±6.13c | 115.94±5.40b | 31.90±3.29a | - | - | R |
| *V. davidi var. nocarpa* LanGao-5 | 15.73±1.48ab | 13.82±1.41a | 34.81±3.74b | 80.08±6.92a | 142.90±11.13a | 20.13±3.40d | + | - | R |
| *V. piasezkii* Liuba-8 | 15.14±1.32b | 13.25±1.28a | 44.49±3.46a | 70.80±3.99b | 140.15±8.17a | 26.86±4.52b | + | + | HR |

**References**

1. Liu R, Wang L, Zhu J, Chen T, Wang Y, Xu Y. Histological responses to downy mildew in resistant and susceptible grapevines*. Protoplas*ma 2015**; 2**52(**1**): 259-270.
